# Occurrence and reproductive roles of hormones in seminal plasma

**DOI:** 10.1186/s12610-017-0062-y

**Published:** 2017-09-08

**Authors:** Jana Vitku, Lucie Kolatorova, Richard Hampl

**Affiliations:** Department of Steroids and Proteofactors, Institute of Endocrinology, Narodni 8, 116 94 Prague, Czech Republic

**Keywords:** Hormones, Steroids, Reproductive hormones, Seminal fluid, Seminal plasma, Spermatogenesis, LC-MS, GC-MS, Immunoassay, Hormones, Stéroïdes, Hormones reproductives, Liquide séminal, Spermatogenèse, LC-MS, GC-MS, Dosage immunologique

## Abstract

Only 2–5% of seminal fluid is composed of spermatozoa, while the rest is seminal plasma. The seminal plasma is a rich cocktail of organic and inorganic compounds including hormones, serving as a source of nutrients for sperm development and maturation, protecting them from infection and enabling them to overcome the immunological and chemical environment of the female reproductive tract. In this review, a survey of the hormones found in human seminal plasma, with particular emphasis on reproductive hormones is provided. Their participation in fertilization is discussed including their indispensable role in ovum fertilization. The origin of individual hormones found in seminal plasma is discussed, along with differences in the concentrations in seminal plasma and blood plasma. A part of review is devoted to methods of measurement, emphasising particular instances in which they differ from measurement in blood plasma. These methods include separation techniques, overcoming the matrix effect and current ways for end-point measurement, focusing on so called hyphenated techniques as a combination of chromatographic separation and mass spectrometry. Finally, the informative value of their determination as markers of male fertility disorders (impaired spermatogenesis, abnormal sperm parameters, varicocele) is discussed, along with instances where measuring their levels in seminal plasma is preferable to measurement of levels in blood plasma.

## Résumé

Les spermatozoïdes ne représentent que 2 à 25% du liquide séminal, le reste étant constitué par le plasma séminal. Le plasma séminal est un cocktail de composés organiques et non organiques comprenant des hormones qui font office de source de substances nutritives pour le développement et la maturation des spermatozoïdes, qui les protègent de l’infection et leur permettent de surmonter l’environnement immunologique et chimique de l’appareil reproducteur féminin. La présente revue propose une vue d’ensemble des hormones retrouvées dans le plasma séminal de l’homme, l’accent étant particulièrement mis sur les hormones reproductives. La participation de ces dernières au processus de fécondation est discutée, y compris leur rôle indispensable dans la fécondation de l’ovocyte. L’origine de chacune des hormones retrouvées dans le plasma séminal est décrite, ainsi que les différences de leurs concentrations dans le plasma séminal et dans le plasma sanguin. Une partie de cette revue est dévolue aux méthodes de mesure, en soulignant des exemples particuliers où elles diffèrent des mesures dans le plasma sanguin. Ces méthodes comprennent les techniques de séparation, qui surmontent les effets matriciels et les procédures actuelles de critère de mesure, en se concentrant sur les techniques dites de couplage comme la combinaison de la séparation chromatographique et de la spectrométrie de masse. Enfin, la valeur informative de la détermination de ces hormones en tant que marqueurs des anomalies de la fertilité masculine (spermatogenèse altérée, paramètres spermatiques anormaux, varicocèle) est discutée, ainsi que les situations où la mesure de leurs taux dans le plasma séminal est préférable à celle du plasma sanguin.

## Background

### The role of seminal plasma in reproduction

Only 2–5% of the seminal fluid is composed of spermatozoa, while the rest is seminal plasma. Seminal plasma acts as a transport medium enabling the penetration of a spermatozoon into the ovum at conception. It provides several indispensable roles: First it serves as a nutritive source for the spermatozoa during their journey through the female reproductive tract. Secondly, it protects from infection and other injurious and toxic agents. Finally, due to its unique composition, it enables spermatozoa to overcome the hostile chemical and immunological milieu of the vagina. The normal vaginal environment is acidic, caused by lactic acid produced by the native microflora. Seminal plasma on the contrary contains basic amines (e.g. putrescine, spermine, spermidine and cadaverine), serving as a buffer to neutralize vaginal acidic conditions. In addition, the vaginal environment is rich in immune cells, the effect of which may be compensated for by various immuno-active molecules from the seminal plasma such as prostanoids (prostaglandins, leukotriens) [1], cytokines [2, 3] and last but not least glucocorticoids [4]. Since sperm cells carry genetic information, it is very important to protect their DNA from acidic denaturation.

To fulfil all these tasks the seminal plasma contains a complex array of organic and inorganic constituents. A large portion consists of nutritive substrates, primarily fructose, citric acid, lactic acid, amino acids and other precursors of main metabolic pathways. There are more than 200 proteins, especially enzymes (such as various proteases, phosphatases etc.), as well as phospholipids, vitamins, antioxidants, and inorganic ions.

Hormones of all kinds are also found among the constituents of seminal plasma. Here we focus on steroids and other reproductive hormones, and their roles. A list of steroids found in human seminal plasma with average concentrations as measured by various authors and methods is shown in Table 1. For comparison, when available, we provide their corresponding blood levels as measured in our laboratory. The collected data from various authors until roughly 2011 were published under Reference [5]. The most striking differences in the ranges of reported physiological values may be seen in the cases of testosterone, androstenedione and especially estradiol. These discrepancies may be at least partially ascribed to different methodologies, based mainly on immunoassays or more or less advanced chromatographic techniques.

**Table 1:** Levels of hormonal steroids and some of their precursors and metabolites in human seminal plasma and blood serum of fertile men

Steroid	Seminal plasma concentration	Plasma/serum concentration	References
Androstanediol (5α-androstane-3α17β-diol)	0.21–1.25		[9]
Androstenedione (4-androstene-3,17-dione)	0.30–2.00	3.0–5.0	See Ref. [5]
	1.20–2.96		[9]
	0.056 (0.035–0.087)	2.036 (1.857–2.228)	[103]
	3.65 ± 1.63		[128]
Cortisol	59–176	140–690	See Ref. [5]
	13 (10–15)	292 (267–320)	[103]
Cortisone	20.6 (18.8–22.4)	71.6 (68.0–75.5)	[103]
Dehydroepiandrosterone (DHEA)	23.4 ± 10.9	24.3 ± 10.3	[14]
	4.9 (3.9–6.1)	14.9 (13.2–16.8)	[103]
Dehydroepiandrosterone sulfate (DHEAS)	1400	6850^a^	See Ref. [35]
Dihydrotestosterone	1.1–1.9	0.87–2.6	See Ref. [5]
	0.775 (0.634–0.933)		[103]
Estrone	0.016 (0.014–0.018)	0.085 (0.070–0.100)	[103]
Estradiol	0.2–0.6	below 0.18	See Ref. [5]
	0.015–0.066		[9]
	0.256 ± 0.073		[40]
	0.242 ± 0.055	0.086 ± 0.023	[65]
	0.070 ± 0.009		[101]
	0.014 (0.012–0.017)	0.062 (0.051–0.077)	[103]
	0.596 ± 0.193	0.095 ± 0.04	[116]
Estriol	0.149 (0.114–0.191)	0.031 (0.021–0.049)	[103]
7α-Hydroxy-DHEA	1.67 ± 0.66	1.41 ± 0.77	[14]
	0.903 (0.782–1.045)	0.913 (0.801–1.041)	[103]
7β-Hydroxy-DHEA	1.45 ± 0.67	1.23 ± 0.60	[14]
	0.210 (0.174–0.246)	0.489 (0.437–0.545)	[103]
16α-Hydroxy-DHEA	1.06 ± 0.15	0–1.86	[15]
17-Hydroxy pregnenolone	0.358 (0.271–0.463)	4.933 (4.241–5.715)	[103]
17α-Hydroxyprogesterone	1.62 ± 1.26	1.30–5.40^b^	[128]
7-oxo-DHEA	0.116 (0.089–0.145)	0.129 (0.106–0.159)	[103]
Pregnenolone	0.626 (0.537–0.730)	1.138 (0.916–1.390)	[103]
Progesterone	1.43 ± 0.56	0–3.20^b^	[128]
Testosterone	0.3–4.6	10–35	See Ref. [5]
	1.18–8.32	10.0–32.2	[9]
	0.07 (0.04–0.11)	11.5 (10.6–12.4)	[103]

The concentration ranges or means ± S.D. or means with 95.0% confidence intervals in parenthesis in nmol/L are shown

^a^Strongly dependent on age, ^b^Data from author’s laboratory

In this review the selection of the literature on hormones and in particular steroids in human seminal plasma is provided, using various combinations of key words as seminal plasma, hormone, steroid, androgens, estrogens, origin etc. Thereafter the most relevant reviews were retrieved and the principle original papers cited.

## The role of sex steroids in ovum fertilization

Steroids, in concert with other components of the seminal plasma as well as from the fluid of the female reproductive tract, influence the process of penetration of the sperm into the ovum. It includes events known as capacitation of spermatozoa and the final penetration into the ovum by release of proteolytic enzymes (“acrosome reaction”). The most effective is progesterone, which is abundant in the fluid of the female reproductive tract. Through its putative membrane receptors on the human spermatozoon membrane progesterone triggers a cascade of rapid non-genomic effects such as a calcium influx, the tyrosine phosphorylation of sperm proteins, a chloride efflux and an increase of cAMP, finally resulting in activation of spermatozoa by the induction of capacitation, increased motility and the activation of proteolytic enzymes responsible for penetration of the sperm across the ovum membrane [6, 7]. However, progesterone is not the only steroid hormone modulating the acrosome reaction. Of interest may be the finding that cholesterol, the precursor of all steroid hormones, may act as an inhibitor of the progesterone effect [8].

Besides progesterone, other steroid hormones are present in both the female reproductive tract and in seminal plasma, and contribute to modulation the above processes [9]. Some in vitro studies have been undertaken to understand the effects of steroid sex hormones: human spermatozoa were incubated with testosterone [10] or estradiol [11], and the acrosome reaction was assessed by staining with Hoechst 33,258 and fluorescein isothiocyanate-conjugated *P. sativum* agglutinin lectin. While no convincing effect of testosterone was found [10], estradiol acted as an inhibitor [11].

## Other steroids in seminal plasma

In addition to the male and female sex steroids, many other hormonal steroids and their precursors and metabolites have been detected in seminal plasma (Table 1). Their concentrations are in most instances (but not always) lower than in blood, due to their passage through accessory sex organs or their in situ biosynthesis. Interestingly, the ratio of biologically active hormones to their inactive counterparts (cortisol/cortisone, testosterone/androstenedione, estradiol/estrone), reflects the activity of steroid metabolizing enzymes. These enzymes include e.g. 11β-hydroxysteroid dehydrogenase of both isotypes in semen [12] and 17β-hydroxysteroid dehydrogenase [13] in spermatozoa. Their eventual role in maintaining an optimal seminal environment is not clear, but measurement of the enzyme activity in ejaculate may be useful for the diagnostics of male reproductive disorders.

With respect to the immune cells patrolling the female reproductive tract, besides prostanoids and related immunoactive molecules, the seminal plasma contains steroids with immunomodulatory properties. Both immunosuppressive cortisol and immunoprotective dehydroepiandrosterone (DHEA) and even its 7-oxygenated metabolites, believed to be the truly active DHEA metabolites, have been found in seminal plasma [14]. Another DHEA metabolite - 16α-hydroxy-DHEA - was hypothesized a counter regulatory steroid to 7-oxygenated steroids [15].

One of the substantive functions of the seminal fluid is also maintaining the electrolyte balance. From this point of view, the finding of an intrinsic renin-angiotensin system in the epididymis and some of it components also in testis, prostate and even in semen is of interest [16]. Information about aldosterone concentrations seems to be lacking in the literature.

Vitamin D (VD) and its active metabolite 1,25-dihydroxycholecalciferol are among the major hormones responsible for calcium homeostasis. The influx of Ca^2+^ participates in the acrosome reaction and calcium is present in remarkable concentrations in seminal plasma [17], the detailed mechanism was studied recently [18]. Human spermatozoa contain all the necessary machinery for its actions, including the VD receptor and VD metabolizing enzymes, the expression of which in human spermatozoa serve as positive predictive markers of sperm quality, with both genomic and non-genomic actions operating there [19–21]. Since both VD receptors and estradiol receptors are present in spermatozoa, an interaction between estrogen and VD signalling occurs there, as shown in a recent review [22]. Surprisingly, there seems to be no data on VD concentrations in seminal plasma, in contrast to numerous reports on blood serum levels [23], though it might be an interesting marker of male reproductive function.

## Sources of steroids in seminal plasma

**Fig. 1:**
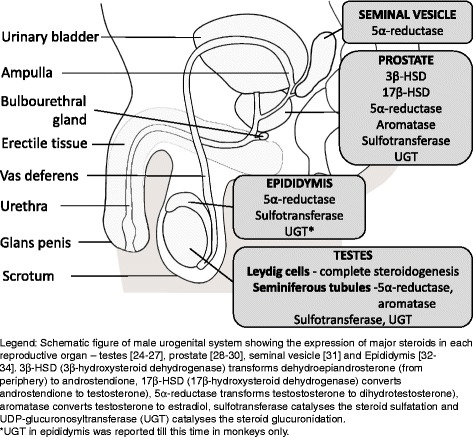
Expression of major steroidogenic enzymes in human reproductive organs

The source of most of the organic as well inorganic constituents in seminal plasma is not the testes but rather accessory sexual organs, namely seminal vesicles, the prostate, and the bulbourethral glands. During emission phase of ejaculation, part of spermatozoa from epididymis and epididymal fluid passes through the vas deferens and the ejaculatory duct –on each side- to arrive in the prostatic urethra where spermatozoa are mixed with fluid form the prostate and the seminal vesicles. In subsequent expulsion phase, spermatozoa and secretions of the previous glands are mixed with the secretions of the bulbourethral glands. Although the main source of major sex steroids are testicular Leydig cells, the male sexual organs also express the major steroidogenic enzymes as shown in Fig. 1 [24–34].

The fact that steroid concentrations in seminal plasma differ from, and in many instances do not even correlate with, their blood levels, indicates their different origin [35]. This primarily concerns the major male sex steroid, testosterone [36]. Testosterone is produced by Leydig cells and is secreted into blood and lymph. Since testosterone is necessary for spermatogenesis, it should be reabsorbed by seminiferous tubules where it must overcome hematotesticular barrier, the permeability of which differs for various steroid hormones [37, 38]. The seminiferous tubule fluid containing spermatozoa is on its route further enriched by other components from epididymis, seminal vesicles and prostate. Since the latter are also target for androgens, it may be supposed that a portion of testosterone and dihydrotestosterone measured in seminal plasma comes from these glands [39]. When compared with blood serum levels, concentrations of testosterone in seminal plasma are lower by almost by one order of magnitude, while dihydrotestosterone and progesterone concentrations are within the range similar to those in blood (Table 1).

Estradiol is the only sex steroid with concentrations in some instances higher in seminal plasma than in blood. This reflects the fact that it is synthesized in the male reproductive system by at least three cell types – Leydig and Sertoli cells and in ejaculated spermatozoa, which, in addition possess a high aromatase activity [5, 40].

## Non-reproductive hormones in seminal plasma

Seminal plasma contains a number of protein/peptide hormones and steroids, as well as some small-molecule hormones such as catecholamines or melatonin. An alphabetical list with the hormone concentrations in seminal plasma and blood plasma/serum (if provided) is shown in Table 2.

**Table 2:** Non-steroidal hormones and their seminal plasma concentrations in fertile/normozoospermic men

Hormone	Seminal plasma concentration	Plasma/serum concentration	References
Adrenalin (pg/mL)	Not detectable - 3028	Not detectable	[69]
Adrenomedulin (pg/mL)	209 ± 19^a^	23.3 ± 2.7^a^	[81]
Antimullerian hormone – AMH (ng/mL)	1.40 ± 0.22^a^		[55]
	<0.49–76.02	<0.49–0.38	[56]
	34.86 ± 23.48		[57]
	5.81 ± 1.526		[58]
	0.098–84.7		[59]
	0.42–49.92		[61]
	0.76 (0.24–10.92)^b^		[62]
	0.34–78.18	0.44–31.89	[64]
Calcitonin (pg/mL)	1771 ± 612		[73]
	1980 ± 521	67 ± 13.1	[74]
	327 ± 25		[76]
	6846.9 ± 3366.4		[77]
	2367 ± 78	30 ± 1.9	[80]
Follicle stimulating hormone - FSH (mIU/mL)	4.6 ± 1.9	9.7 ± 6.7	[43]
	3.5 ± 2.03	5.3 ± 2.2	[44]
Human chorionic gonadotropin - hCG (ng/mL)	1.87 ± 0.93		[53]
	3.73 ± 1.60		[54]
Inhibin B (pg/mL)	714.36 ± 522.66		[59]
	7.8–9874	54.68 ± 70.85	[61]
	44.0 (20.7–200.3)^b^		[62]
Luteinizing hormone – LH (mIU/mL)	19.4 ± 9.9	13.6 ± 8.5	[43]
Melatonin (pg/mL)	44.75 ± 11.0	111.75 ± 35.3	[44]
	1.7 ± 1.0	3.1 ± 1.4	[65]
	23.7 ± 10.9^a^		[68]
Noradrenalin (pg/mL)	15,181 ± 2951^a^	501 ± 136.5	[69]
Oxytocin (pg/mL)	1.72 ± 0.78		[49]
	61.1 ± 11.7		[51]
Parathormone (pg/mL)	2846 ± 611.6		[72]
Prolactin (PRL) (ng/mL)	3.5 ± 0.85	6.4 ± 0.85	[44]
	46.6 ± 2.0^a^	17.8 ± 1.7^a^	[46]
	43.2 ± 2.7^a^	6.2 ± 0.7^a^	[47]
	133.6 ± 47	24.5 ± 8	[48]
Vasopressin (antidiuretic hormone – ADH) (pg/mL)	1.84 ± 1.23		[52]

Values are expressed as means ± standard deviations or concentration ranges. Information on blood plasma/serum concentrations is also provided if listed in the article

^a^mean ± standard error

^b^median (interquartile range)

**Gonadotropins**, luteinizing hormone (LH), follicle stimulating hormone (FSH) and **prolactin** were detected in human seminal plasma as early as the 1980s. They are believed to be transported from the blood to semen via accessory sex organs. Levels of FSH are slightly lower and on the contrary LH levels are slightly higher but within the range of those in serum [41–44]. Prolactin is mostly reported to be higher in seminal plasma [45–48].

Hypothalamic nonapeptides, **oxytocin and vasopressin** (an antidiuretic hormone - ADH) were found in human seminal plasma in the 1990s in amounts in the low pg/mL range. Circulating hormones stem from the hypothalamus and are released by neurohypophysis, but there are also other peripheral sources of these hormones, especially the prostate [49–52].

Alpha and beta subunits of **human chorionic gonadotropin** (hCG) have been studied in human seminal plasma over the past 30 years [41, 53, 54]. The levels of beta-hCG in seminal plasma were found to be higher than in the serum and to correlate with sperm parameters [53, 54].

**Antimüllerian hormone (AMH) and inhibin(s).** Quite a bit of attention has been paid to seminal AMH [55–64] and to a lesser extent to inhibin B, important peptides for male as well as female reproduction [59, 61]. The first report on AMH in seminal plasma dates from 1996 [55]. The range of physiological AMH concentrations in seminal plasma is reported to be very large – almost two orders of magnitude [56, 59, 61, 62, 64]. It is believed that both hormones well reflect sperm production and the development of Sertoli cells [57] and concentrations of both in seminal plasma are higher than in serum [60].

**Melatonin**, a small molecule produced by the pineal gland, has been also detected in seminal plasma, but its concentrations were approximately one order of magnitude lower than in blood [44, 65]. Its presence in seminal plasma is important with respect to its antioxidant capacity as a radical scavenger [66, 67]. Lower levels of both seminal and blood melatonin have repeatedly been found in men with impaired spermatogenesis compared to healthy fertile men [44, 68]. Furthermore, melatonin has been suggested as a therapeutic for the improvement of sperm motility in assisted reproduction [67].

**Catecholamines**. There is surprisingly little information about catecholamines in seminal plasma, though they are present in considerable concentrations exceeding the levels in blood plasma. Major catecholamines – noradrenaline and adrenaline and their precursors - 3,4-dihydroxy-phenylalanine (DOPA), and 3,4-dihydroxy-phenyl acetic acid (DOPAC) were measured in seminal plasma from healthy men by HPLC as early as in 2001 [69]. Their role is still the subject of debate. It has been suggested that they participate in immunological processes enabling the penetration of sperm into the female reproductive tract, specifically participating in a local adaptive shift in the balance of T helper lymphocytes (Th) to a dominance of Th2 in the maternal tract [70, 71]. However, no correlation has been found between concentrations of any of the catecholamines and semen characteristics [69].

**Parathormone and calcitonin**. Both of these calcium regulatory peptide hormones have been detected in human seminal plasma [72–80], to which they enter from accessory sex organs, especially the prostate [78]. From the point of view of their role in male reproduction, calcitonin is the more important, and concentrations in the semen are 30 times higher than in blood plasma [74]. No differences, however, have been found between fertile and infertile men [76], or between normo-, oligo-, or even azoospermic men [77]. The only finding has been an association of lower calcitonin content in patients with decreased sperm motility [80]. It was demonstrated, that seminal calcitonin participates in calcium regulation during the process of sperm capacitation [79].

**Adrenomedullin**. The recently discovered vasoactive peptide adrenomedullin has also been detected in seminal plasma. High levels of this hormone have been associated with decreased sperm counts, but its role in the regulation of male fertility remains unknown [81].

**Thyroid hormones**. Though thyroid status is important for male fertility (see e.g. Refs [82, 83]), reports on thyroid hormones in seminal plasma seem to be lacking.

## Methods for the determination of steroids in seminal plasma

Since the 1970s, steroid hormones started to be measured mainly in blood, urine, and saliva, but also in seminal plasma. The main technique used became radio- and other immuno-assays, because in comparison with former analytical techniques they provided a million-fold increase in sensitivity. Later separation techniques combined with mass spectrometry methods were developed, and brought even greater sensitivity to steroid assessments.

Human semen coagulates immediately after ejaculation followed by liquefaction that occurs within 20 min. Ejaculate liquefies due to proteolytic fragmentation of mainly semenogelins (Semenogelin I and II) [84, 85] and fibronectins [86]. Some of semen samples fail to liquefy and remain highly viscous which can indicate the disorders of accessory glands function. High viscosity can interfere with determination of some biochemical markers [87]. Whereas some of proteomic studies promptly centrifuge the sample prior liquefaction to avoid proteolysis together with adding proteases inhibitors, seminal plasma for steroid analysis is left to liquefy and subsequently undergo centrifugation to gain supernatant – seminal plasma - which is further processed or stored in −20 °C (or −80 °C) until analysis.

### Techniques for steroid extraction and sample processing

Many laboratories performing steroid assays employ liquid-liquid extraction (LLE) as an initial step in the purification and concentration of steroids of interest. Eventual binding to transport proteins can be completely eliminated by extraction to an organic solvent. The most common solvents used in LLE are methyl *tert*-butyl ether, diethyl ether, ethyl acetate, dichloromethane or mixtures of organic solvents [88]. These might be useful in reducing matrix effects, since ionized compounds, such as salts or phospholipids, do not partition into the organic layer [89]. It is important note that steroids often bind very tightly to glass. This fact has to be taken into account when developing and optimizing a method. Today, immunoassay kits often offer direct analysis without an extraction step, allowing faster analysis. Since the composition of seminal plasma and blood plasma may differ considerably, before using kits designated for blood plasma measurements they should be first validated for seminal plasma.

Chromatographic techniques usually require an extraction step as well. Apart from LLE, other possibilities for steroid sample preparation include on-line or off-line solid-phase extraction (SPE) [90] and supported liquid extraction (SLE) [91]. Compared with off-line SPE, on-line SPE is advantageous in that several steps in sample preparation are eliminated, and the automation results in better repeatability and reproducibility [92]. Technique of SLE is relatively new in steroid hormone analysis and compared to SPE, SLE included fewer steps in protocol and thus was less time consuming and potentially also cost-effective [91]. However, we know of no studies on either off-line or on-line SPE or SLE employed in assessments of seminal plasma steroids. When using gas chromatography-mass spectrometry (GC-MS), attention should be paid to any plastic material used during the sample preparation, since many plastics contain phthalates that can interfere with the final analysis.

### Separation techniques and hyphenated techniques

Prior to immunoassays, paper chromatography [36], thin layer chromatography [93], column chromatography [94–97] and high performance liquid chromatography (HPLC) [14, 98] have been used to purify samples. These separation techniques also allow the assessment of more analytes from one sample, even if the concentrations of analysed steroids are low.

In recent years, on-line combinations of a separation technique and one or more spectroscopic detection techniques have received increasing attention, and have been termed hyphenated techniques. Liquid chromatography and gas chromatography coupled with mass detector (LC-MS and GC-MS, respectively) have become the preferred approach in steroid analysis [99]. Surprisingly, as far as we know only two groups have used hyphenated systems for determinations of steroids in seminal plasma [15, 100–103], but they assessed a broad spectrum of seminal steroids - pregnenolone, 17-hydroxy-pregnenolone, cortisol, cortisone, DHEA, 16α-hydroxy-DHEA, 7α-hydroxy-DHEA, 7β-hydroxy-DHEA, 7-oxo-DHEA, testosterone, androstenedione, dihydrotestosterone, estrone, estradiol and estriol.

### Mass spectrometry, immunoassays and their strengths and weaknesses

Some methods for steroid determinations use an extraction and/or chromatographic step prior to end point measurements (mass spectrometry, immunoassay). However, mass spectrometry (MS) methods often allow measurement without an extraction step, and provide sufficient sensitivity despite the complex matrix. On the other hand, if the sensitivity needs to be enhanced, derivatization of steroids is a further possibility (reviewed in [104]). Estrogen phenyl groups are commonly derivatized by dansyl chloride in acetone [100, 105, 106], on the other hand 2-hydrazino-pyridine in methanol is very effective in enhancing sensitivity of oxo as well as di-oxosteroids [103, 107, 108].

It is not uncommon in immunoassays to report higher analyte concentrations in comparison with LC-MS or GC-MS systems (e.g. [109–111]). Chromatography - mass spectrometry systems typically measure only a single compound (on one transition), while antibodies used in immunoassay sometimes recognize not only the target molecule but also structurally related molecules. In addition to endogenous structurally related molecules, some drugs (such as anabolic steroids and herbal medications) and natural products can cross-react with the antibody and thus increase the apparent analyte concentration [112].

Apart the fact that seminal sampling is non-invasive, seminal plasma is a more “suitable” matrix for immunoassays as it does not contain the common interferences present in blood plasma such as hemolysis, icterus or lipemia. Lipid levels are significantly lower in seminal plasma [113]. However, the seminal plasma proteome is as complex as the proteome of blood plasma (for review see [114]), and proteins like albumin and mucin can also interfere with the immunoassay [115].

The most noticeable difference between concentrations measured by immunoassays and MS technique is for estradiol. When a chromatographic step has been used before final measurements, estradiol levels have been reported in the low pg/mL range (approximately to 20 pg/mL) [9, 100–102], which is lower than in blood plasma. Immunoassays of estradiol in the seminal plasma of healthy men have given results, e.g., of 65.9 ± 15.0 pg/mL [65], 69.7 ± 20.0 pg/mL [40] and 162.4 ± 52.5 pg/mL [116], which are concentrations higher than in blood plasma. This discrepancy can be explained by the cross-reactivity of antibodies with other steroids such as estrone, estriol and conjugated estrogens all of which are present in seminal plasma in higher concentrations than unconjugated estradiol [101, 102]. The results of our laboratory have shown that estrone is present in slightly higher concentration in seminal plasma as estradiol, and estriol is even 20 times higher [102]. The second reason may be the use of commercial kits that were not validated for seminal matrix. Furthermore, the limits of detections of assay kits are often higher than the estradiol levels measured by MS methods. Nevertheless, all studies have agreed that higher levels of seminal estradiol are found in men with various degree of infertility in comparison with healthy men [9, 40, 102, 116, 117].

Matrix effects have been considered as the Achilles heel in LC-MS analysis [118]. To assess the matrix effects, three different strategies currently exist: (1) post-column infusion, (2) post-extraction addition, and (3) a comparison of the slopes of calibration curves [88]. Validation of analytical methods and evaluations of matrix effects in seminal plasma are more complicated because there is not as much seminal fluid material in comparison with e.g. blood plasma, and seminal plasma stripped of steroids is not commercially available. In blood plasma, charcoal-stripped serum is usually used for the preparation of calibration curves and quality controls. However, components of actual samples that cause matrix effects can be removed by the charcoal stripping process [88]. Modification of the sample extraction procedure and improving the chromatographic separation are essential in minimizing the matrix effects [118]. The addition of isotope-labelled internal standards at the beginning of sample preparation can be used to compensate for alterations in the signal [119].

## Using seminal hormones as diagnostic and prognostic tools in male fertility disorders

The first reports on the determination of hormones in the seminal plasma appeared as early the late 1970s (for a review of the literature see Refs. [5, 35, 40]). Initially, the main focus was on how seminal hormone concentrations correlated with sperm parameters (sperm count, motility, percentage of damaged sperms etc.) and with the respective blood plasma levels. Later reports focused on more the detailed forms and causes of fertility disorders (severity of oligozoospermia, combinations with other sperm disorders such as oligoasthenozoospermia, oligoasthenoteratozoospermia and even azoospemia) [9, 40]. Here only those hormones which may serve as markers of male fertility disorders are mentioned.

**Sex steroids**: The majority of studies so far have dealt with sex steroids. From the data available the following conclusions may be drawn: men with impaired spermatogenesis as given by their total sperm count, decreased motility and increased percentage of morphologically altered spermatozoa had generally lower seminal concentrations of dihydrotestosterone [36, 103] and androstenedione [9] in comparison with healthy fertile men. Their levels of estradiol [9, 40, 102, 103, 116, 117], other estrogenic steroids [103], DHEA [103], 5α-androstane-3α17β-diol [9], progesterone [9] and 17α-hydroxyprogesterone [9] were increased. As for testosterone, while in most earlier reports authors did not find significant differences between healthy men and those with impaired spermatogenesis, more recent refinements of analytical methods have revealed lower seminal testosterone in oligo-, astheno- or azoospermic men (for review see [5, 40]). These results have been confirmed by the recent paper of Zalata et al. [9], who also studied the effect of a varicocele in oligoasthenoteratozoospermic men, but did not find any difference between men with or without this urogenital disorder.

In conclusion, many reviews have looked for associations of serum steroid and other hormones levels with impaired spermatogenesis, but not always with definite results (see e.g. [120, 121]). This raises the question of what advantage (if any) are determinations of seminal steroids over blood plasma analysis. Our evaluation of the available data suggests that primarily seminal dihydrotestosterone and the testosterone/estradiol ratio may be useful [5, 40].

**Cortisol**: There are only a few reports concerning seminal cortisol (see Refs [4, 103]), though this steroid is known to affect negatively testosterone production in Leydig cells. An important counter-regulatory mechanism in these cells consists of the oxidation of excessive cortisol by 11β-hydroxysteroid dehydrogenase (11β-HSD) type 2. Activities of this enzyme have been measurable in semen, although seminal plasma alone was devoid of 11β-HSD activity [12]. Cortisol along with its precursors progesterone and 17β -hydroxyprogesterone have been detected in considerable amounts in samples of sonicated specimens of sperm obtained as ejaculates from husbands of infertile couples, and their levels correlated with sperm count [122]. More studies are needed for an evaluation of cortisol and its precursors in seminal plasma as potential markers of impaired spermatogenesis.

**LH, FSH and prolactin**: Following their detection in seminal plasma, the levels of gonadotropins and prolactin have been compared in fertile and infertile men. Though generally lower in infertile groups, their measurement in seminal plasma did not contribute to improvements in the diagnosis of infertility in comparison with blood plasma [41, 42, 45]. On the contrary, one promising marker of male fertility disorders may be the free beta subunit of human chorionic gonadotropin [53].

**Oxytocin and vasopressin**: More attention has been paid to oxytocin than vasopressin due to its potential effect on sperm transport as measured by their motility. While the first report did not find any relationship between oxytocin seminal plasma levels and sperm characteristics [49], a more recent study on infertile men with varicocele revealed a significant negative correlation of seminal oxytocin with sperm count and motility, and a significant positive correlation with the percentage of abnormal sperm forms. Moreover, seminal oxytocin has been associated with varicocele grade and its bilaterality [51].

**AMH and inhibin(s)**: In spite of their wide range of physiological concentrations, it appears that AMH and inhibin B positively correlate with parameters of sperm quality such as sperm count and motility, and negatively with the percentage of damaged spermatozoa [56, 57, 64]. AMH in seminal plasma was not detectable in obstructive azoospermia but it was proposed as a good marker for hypospermatogenesis in cases of non-obstructive azoospermia [56]. AMH and inhibin B have been further tested as for their predictive value for outcomes of testicular sperm extraction. It was concluded, however, that either alone or in combination they are poor predictors for this purpose [59, 61]. On the other hand AMH and inhibin B may be successfully used for predictions of motile sperm recovery after semen cryopreservation [62]. Seminal AMH is also a good marker for assessments of recombinant FSH treatment in men with idiopathic infertility undergoing assisted reproduction cycles [63].

Seminal plasma contains a wide range of protein molecules as well. Proteomics expanded significantly over the past decade, which correlates with better analytical instrumentation and methodologies. Mass spectrometry - based proteomics is now promising tool in searching for protein biomarkers of male infertility and pathologies of male reproductive tract. Recent study of Rolland et al. determined several protein biomarkers specific to each organ of male reproductive tract that could be used in diagnostics of male infertility, especially in non-obstructive azoospermia [123]. The another study identified two protein biomarkers (ECM1 and TEX101) that can distinguish between non-obstructive and obstructive azoospermia with high sensitivity and specificity [124]. The latest analytical techniques enable measurement of post-translationally modified proteins – such glycoproteins in human seminal plasma [125] of which sialylated fibronectin was found to be associated with abnormal sperm parameters [126, 127].

## Conclusion

The determination of hormones and especially reproductive hormones in seminal plasma is an important tool for the diagnostics and treatment success of male fertility disorders, and in some instances is to be preferred over determinations in blood plasma. Modern analytical approaches enable the measurement of a wide array of hormones including steroids and peptide hormones, with better sensitivity and limits of detection.

## Abbreviations

11β-HSD: 11β-hydroxysteroid dehydrogenase
ADH: Antidiuretic hormone
AMH: Antimüllerian hormone
DHEA: Dehydroepiandrosterone
DNA: Deoxyribonucleic acid
DOPA: 3,4-dihydroxy-phenylalanine
DOPAC: 3,4-dihydroxy-phenyl acetic acid
FSH: Follicle stimulating hormone
GC-MS: Gas chromatography-mass spectrometry
hCG: Human chorionic gonadotropin
HPLC: High performance liquid chromatography
LC-MS: Liquid chromatography-mass spectrometry
LH: Luteinizing hormone
LLE: Liquid-liquid extraction
MS: mass spectrometry
PRL: Prolactin
SLE: supported liquid extraction
SPE: Solid-phase extraction
Th: T helper lymphocytes
UGT: UDP-glucuronosyltransferase
VD: Vitamin D
